# Supplementary material for: Characterization of a Preclinical In Vitro Model Derived from a SMARCA4-Mutated Sinonasal Teratocarcinosarcoma
Source: Cells. 2023 Dec 30;13(1):81. doi: 10.3390/cells13010081 (PMC10778008; doi:10.3390/cells13010081)
Supplement: Supplementary file 1 [file cells-13-00081-s001.zip › cells-2770860-supplementary.pdf]

Supplementary data

Characterization of a preclinical in vitro model derived from a SMARCA4-mutated sinonasal teratocarcinosarcoma.

Sara Lucila Lorenzo-Guerra1, Helena Codina-Martínez1, Laura Suárez-Fernández1, Virginia N. Cabal1, Rocío García-Marín1, Cristina Riobello1, Blanca Vivanco2, Verónica Blanco-Lorenzo2, Paula Sánchez-Fernández3 Fernando López3, Jóse Luis Llorente3, Mario A. Hermsen1

Table S1. Cell line authentication. The identity of cell line TCS627 was confirmed by comparing the STR profile to the primary tumour to normal blood lymphocytes of the patients from which the cell line was derived. The Promega Powerplex 16 system (Promega Biotech Ibérica SL, Barcelona, Spain) was used to amplify fifteen STR loci and the non-microsatellite, gender-specific amelogenin locus.

Chromosome	STR marker	Blood lymphocytes	Primary tumour	TCS627
Xp	AMEL	X Y	X Y	X Y
5q	CSF1PO	12	12	12
13q	D13S317	13 11	13 11	13 11
16q	D16S539	13 8	13 8	13 8
18q	D18S51	13 22	13 22	13 22
21q	D21S11	30 31.2	30 31.2	30 31.2
3p	D3S1358	17 15	18 15	17 15
5q	D5S818	11 12	12	11 12
7q	D7S820	8 11	8 11	8 11
8q	D8S1179	14 13	14 13	14 13
4q	FGA	18 24	18 24	18 24
21q	Penta D	14	14	14
15q	Penta E	11 13	11 13	11 13
11p	TH01	6 8	6 8	6 8
2p	TPOX	11	11	11
12p	vWa	14 18	14 18	14 18

Table S2. A complete description of all WES results of cell line TCS627, its corresponding primary tumor and normal DNA derived from blood lymphocytes.

Gene	Chr	Position	c.Hgvs	p.Hgvs	Protein Effect	Variant reads Normal	Variant reads primary tumor	Variant reads TCS627	Coverage Normal	Coverage primary tumor	Coverage TCS627	Variant Freq Normal	Variant Freq primary tumor	Variant Freq TCS627
A2M	12	9068775	c.4331C>G	p.Pro1444Arg	no_syn	0	55	132	179	237	391	0	0,23	0,34
ACMSD	2	134872633	c.841A>G	p.Ile281Val	no_syn	0	44	43	112	105	106	0	0,42	0,41
ADGRB1	8	142464480	c.282C>A	p.Ser94Arg	no_syn	0	139	47	295	466	173	0	0,30	0,27
ADGRG4	X	136347484	c.3778C>A	p.Gln1260Lys	no_syn	0	72	145	103	98	145	0	0,73	1,00
AP5B1	11	65779771	c.722G>C	p.Ser241Thr	no_syn	0	83	121	258	223	171	0	0,37	0,71
ARHGEF18	19	7375721	c.472C>G	p.Leu158Val	no_syn	0	32	26	93	81	**40**	0	0,40	0,65
ARID2	12	45846872	c.1518_1519del	p.Gln507Alafs*13	frameshift	0	38	73	133	161	184	0	0,24	0,40
ATP13A5	3	193310690	c.2473A>G	p.Arg825Gly	no_syn	0	33	38	90	96	100	0	0,34	0,38
BAALC	8	103212909	c.161-10C>T		splicing	0	92	92	261	366	292	0	0,25	0,32
C3orf84	3	49177868	c.383T>C	p.Ile128Thr	no_syn	0	55	30	159	129	84	0	0,43	0,36
CAB39	2	230760045	c.44T>C	p.Ile15Thr	no_syn	0	37	56	100	101	75	0	0,37	0,75
CACNA1A	19	13359680	c.904G>C	p.Asp302His	no_syn	0	37	44	170	133	153	0	0,28	0,29
CAPN1	11	65185944	c.484G>A	p.Val162Met	no_syn	0	63	54	229	183	172	0	0,34	0,31
CAPN6	X	111263907	c.29delA	p.Asn10Thrfs*7	frameshift	0	45	58	47	55	68	0	0,82	0,85
CARM1	19	10908131	c.439G>T	p.Val147Leu	no_syn	0	50	20	149	141	59	0	0,35	0,34
CAV3	3	8733913	c.37A>G	p.Ile13Val	no_syn	0	126	259	354	336	425	0	0,38	0,61
CDC20	1	43363049	c.1420G>A	p.Glu474Lys	no_syn	0	47	41	133	154	83	0	0,31	0,49
CDCP1	3	45118580	c.124G>C	p.Val42Leu	no_syn	0	43	35	137	116	112	0	0,37	0,31
CDH4	20	61934117	c.2441C>T	p.Pro814Leu	no_syn	0	50	86	140	152	129	0	0,33	0,67
CDHR1	10	84199074	c.391G>C	p.Glu131Gln	no_syn	0	53	57	105	120	130	0	0,44	0,44
CDKN2A	9	21971162	c.223_239dupGGAG CTGCTGCTGCTCC	p.Arg81Glufs*?	frameshift	0	63	40	369	250	104	0	0,25	0,38
COL15A1	9	99003458	c.1071A>T	p.Leu357Phe	no_syn	0	87	53	198	222	116	0	0,39	0,46
COL7A1	3	48581264	c.4895G>A	p.Arg1632Gln	no_syn	0	83	84	241	269	125	0	0,31	0,67
COPS8	2	237094203	c.439+6T>A		splicing	0	66	49	152	156	134	0	0,42	0,37
CPT1A	11	68762674	c.1828A>G	p.Thr610Ala	no_syn	0	75	107	282	215	282	0	0,35	0,38
CRAMP1	16	1656840	c.2083A>G	p.Met695Val	no_syn	0	82	73	189	215	124	0	0,38	0,59
CSNK2A1	20	499907	c.241G>C	p.Glu81Gln	no_syn	0	13	13	56	**32**	**24**	0	0,41	0,54
CYP11B2	8	142914264	c.954G>C	p.Thr318Thr	splicing | syn	0	113	128	194	231	186	0	0,49	0,69
DAB1	1	57015140	c.1187C>T	p.Ser396Phe	no_syn	0	112	129	278	313	253	0	0,36	0,51
DUSP11	2	73780067	c.49T>A	p.Phe17Ile	no_syn	0	93	50	268	255	125	0	0,36	0,40
EVX2	2	176080544	c.994C>T	p.Pro332Ser	no_syn	0	32	27	52	76	**32**	0	0,42	0,84
FAM200A	7	99547050	c.1358A>T	p.Lys453Ile	no_syn	0	21	15	56	62	**40**	0	0,34	0,38
FEZF2	3	62372136	c.733G>A	p.Val245Ile	no_syn	0	82	42	224	203	121	0	0,40	0,35

GABRB3	15	26561045	c.967G>T	p.Glu323*	stop_gained	0	14	0	102	108	106	0	0,13	0,00
HAO1	20	7885741	c.936dupT	p.Val313Cysfs*?	frameshift	0	53	33	190	169	142	0	0,31	0,23
HDLBP	2	241239614	c.2598C>G	p.Ile866Met	no_syn	0	63	104	240	198	158	0	0,32	0,66
HIC2	22	21445181	c.286A>G	p.Ile96Val	no_syn	0	112	103	385	323	339	0	0,35	0,30
HNRNPUL1	19	41302702	c.1725T>G	p.Asp575Glu	no_syn	0	65	78	222	197	130	0	0,33	0,60
IPO7	11	9425245	c.1318G>A	p.Ala440Thr	no_syn	0	10	15	50	**36**	**24**	0	0,28	0,63
ITGAE	17	3751696	c.1847A>G	p.Tyr616Cys	no_syn	0	41	30	135	117	**48**	0	0,35	0,63
JAKMIP1	4	6060471	c.1597C>G	p.Gln533Glu	no_syn	0	38	36	128	124	133	0	0,31	0,27
KCNQ3	8	132137985	c.1599dupA	p.Phe534Ilefs*15	frameshift	0	20	0	69	115	97	0	0,17	0,00
KIF26A	14	104175603	c.2815G>A	p.Ala939Thr	no_syn	0	59	56	117	140	83	0	0,42	0,67
KLHL40	3	42686493	c.875C>T	p.Ala292Val	no_syn	0	74	75	255	235	288	0	0,31	0,26
KPNA6	1	32157430	c.316A>G	p.Lys106Glu	no_syn	0	46	33	120	119	79	0	0,39	0,42
LOC100129307	13	77661672	c.955G>A	p.Gly319Ser	no_syn	0	18	26	141	155	92	0	0,12	0,28
LZTS2	10	101002639	c.101C>G	p.Ser34*	stop_gained	0	97	101	282	259	201	0	0,37	0,50
MACF1	1	39432640	c.11257C>T	p.Leu3753Phe	no_syn	0	16	24	93	52	52	0	0,31	0,46
MALRD1	10	19175221	c.1844C>A	p.Ala615Asp	no_syn	0	29	55	74	89	124	0	0,33	0,44
MAP1A	15	43522580	c.1107G>T	p.Lys369Asn	no_syn	0	51	79	134	114	120	0	0,45	0,66
MAP1LC3B	16	87398836	c.62G>A	p.Arg21Gln	no_syn	0	73	109	186	185	219	0	0,39	0,50
MAP1S	19	17727729	c.2345C>T	p.Thr782Met	no_syn	0	124	52	404	324	164	0	0,38	0,32
MAPK9	5	180280478	c.84G>C	p.Gln28His	no_syn	0	30	78	95	91	112	0	0,33	0,70
MC2R	18	13885517	c.2T>C	p.Met1?	first_met	0	85	89	196	209	253	0	0,41	0,35
MEGF10	5	127402622	c.857G>A	p.Gly286Glu	no_syn	0	76	108	205	207	316	0	0,37	0,34
MFSD12	19	3547907	c.777delT	p.Ala260Profs*26	frameshift	0	66	47	230	179	74	0	0,37	0,64
MICALL2	7	1442193	c.1710G>T	p.Gln570His	splicing | no_syn	0	30	16	86	93	**44**	0	0,32	0,36
MROH5	8	141467400			splicing	0	26	18	68	87	**46**	0	0,30	0,39
NCKAP1	2	183003295	c.268T>G	p.Ser90Ala	no_syn	0	12	24	54	**39**	**45**	0	0,31	0,53
ND1	MT	3697	c.391G>A	p.Gly131Ser	no_syn	0	224	396	187	262	396	0	0,85	1,00
NELL2	12	44875225	c.184G>A	p.Asp62Asn	splicing | no_syn	0	48	77	132	188	214	0	0,26	0,36
NOBOX	7	144399081	c.1241delC	p.Pro414Hisfs*20	frameshift	0	47	93	141	143	169	0	0,33	0,55
NOTCH3	19	15189408	c.1057G>A	p.Asp353Asn	no_syn	0	112	154	343	310	229	0	0,36	0,67
NOVA2	19	45939954	c.1388C>T	p.Pro463Leu	no_syn	0	130	94	412	362	316	0	0,36	0,30
OLR1	12	10166940	c.196C>A	p.Leu66Ile	no_syn	0	50	51	114	95	75	0	0,53	0,68
PHF3	6	63684280	c.558T>A	p.Cys186*	stop_gained	0	56	52	111	134	171	0	0,42	0,30
PKN2	1	88786112	c.1180T>A	p.Cys394Ser	no_syn	0	8	12	46	**33**	**30**	0	0,24	0,40
POU6F2	7	39433137	c.1087A>G	p.Thr363Ala	no_syn	0	140	124	235	296	225	0	0,47	0,55
PTPRA	20	2988080	c.576A>C	p.Leu192Phe	no_syn	0	13	30	54	55	50	0	0,24	0,60
PTPRC	1	198752298	c.3257T>C	p.Ile1086Thr	no_syn	0	47	75	152	182	246	0	0,26	0,30
RNF212B	14	23268956	c.667T>C	p.Ser223Pro	no_syn	0	25	18	94	71	**48**	0	0,35	0,38
SATB2	2	199272684	c.1741-12C>G		splicing	0	21	31	77	75	52	0	0,28	0,60

SBF2	11	9842723	c.3157delA	p.Met1053Termfs*1	frameshift	0	74	102	260	206	279	0	0,36	0,37
SBNO1	12	123302867	c.3799G>C	p.Asp1267His	no_syn	0	33	37	88	92	103	0	0,36	0,36
SCAMP1	5	78450039	c.734+16dupT		splicing	0	5	0	33	**35**	**46**	0	0,14	0,00
SCN3A	2	165147029	c.1381G>C	p.Ala461Pro	splicing | no_syn	0	15	14	55	**47**	**41**	0	0,32	0,34
SEZ6L	22	26365384	c.2612T>G	p.Leu871Arg	no_syn	0	59	96	133	138	149	0	0,43	0,64
SH3PXD2A	10	103603282	c.1852G>A	p.Gly618Ser	no_syn	0	41	0	166	187	170	0	0,22	0,00
SHQ1	3	72844433	c.144-10A>G		splicing	0	22	37	86	51	61	0	0,43	0,61
SHROOM1	5	132825276	c.865T>A	p.Ser289Thr	no_syn	0	109	46	293	298	118	0	0,37	0,39
SLC20A1	2	112660394	c.1615A>G	p.Ile539Val	no_syn	0	39	42	175	113	112	0	0,35	0,38
SLC35E3	12	68752096	c.578C>T	p.Pro193Leu	no_syn	0	25	52	92	91	132	0	0,27	0,39
SLC7A10	19	33211226	c.1015A>G	p.Arg339Gly	splicing | no_syn	0	118	188	377	325	295	0	0,36	0,64
SLCO5A1	8	69673171	c.2245G>A	p.Gly749Arg	no_syn	0	46	35	110	196	104	0	0,23	0,34
SMARCA4	19	10991149	c.1246-1G>C		canonical_splicing	0	68	133	222	203	205	0	0,33	0,65
SMPD4	2	130161269	c.867_868delinsCT	p.Glu290*	inframe	0	17	41	62	76	60	0	0,22	0,68
SMPD4	2	130161269	c.868G>T	p.Glu290*	stop_gained	0	17	41	62	77	60	0	0,22	0,68
SPAM1	7	123954217	c.647G>C	p.Trp216Ser	no_syn	0	27	20	93	94	64	0	0,29	0,31
STAG2	X	124076335	c.2537G>T	p.Gly846Val	no_syn	0	39	53	57	59	53	0	0,66	1,00
STRN4	19	46727963	c.1084G>C	p.Asp362His	no_syn	0	48	86	193	149	130	0	0,32	0,66
TET2	4	105234805	c.863C>T	p.Pro288Leu	no_syn	0	37	69	141	131	177	0	0,28	0,39
TICRR	15	89575822	c.236A>G	p.Glu79Gly	no_syn	0	77	57	185	183	82	0	0,42	0,70
TRHDE	12	72499576	c.1663C>T	p.Gln555*	stop_gained	0	86	109	217	270	560	0	0,32	0,19
UBA1	X	47202496	c.1048C>T	p.Arg350Cys	no_syn	0	148	256	210	186	256	0	0,80	1,00
VWDE	7	12375238	c.1025-11T>A		splicing	0	35	41	69	64	61	0	0,55	0,67
WNT7A	3	13818951	c.1043G>T	p.Cys348Phe	no_syn	0	83	148	175	193	211	0	0,43	0,70
ZC3H12D	6	149474459	c.85G>A	p.Glu29Lys	no_syn	0	50	72	194	165	101	0	0,30	0,71
ZNF2	2	95181784	c.956G>C	p.Gly319Ala	no_syn	0	63	95	161	158	146	0	0,40	0,65
ZNF615	19	52002197	c.100C>T	p.Arg34Trp	no_syn	0	22	142	178	165	220	0	0,13	0,65
